# Predictive Biomarkers in Cancer Immunotherapy: A Narrative Review Across Selected Solid Tumors

**DOI:** 10.7759/cureus.88647

**Published:** 2025-07-24

**Authors:** Mehak Puntambekar, Neville Shery, Ishaan Parokkaran, Mohammed Al-Hamas

**Affiliations:** 1 Medicine and Surgery, Pilgrim Hospital, United Lincolnshire Hospitals National Health Service (NHS) Trust, Boston, GBR; 2 General Medicine, Pilgrim Hospital, United Lincolnshire Hospitals National Health Service (NHS) Trust, Boston, GBR; 3 Emergency Medicine, Pilgrim Hospital, United Lincolnshire Hospitals National Health Service (NHS) Trust, Boston, GBR; 4 Acute Internal Medicine, University of Hull, Hull, GBR

**Keywords:** biomarkers, cancer immunology, ctla-4, immunotherapy, melanoma, msi, nsclc, pd-l1, tumor-infiltrating lymphocytes

## Abstract

Cancer immunotherapy has transformed the therapeutic landscape for several malignancies, offering durable responses in select patient populations. However, response rates remain variable, underscoring the need for robust predictive and prognostic biomarkers. This narrative review synthesizes current evidence on biomarkers guiding immunotherapy in non-small cell lung cancer (NSCLC), melanoma, breast cancer, and colorectal cancer. Literature was identified through manual screening of PubMed and Scopus using key terms related to immunotherapy biomarkers, with no date restrictions applied; studies published up to May 2025 were included.

Clinically validated markers such as programmed death-ligand 1 (PD-L1) and microsatellite instability-high (MSI-H) demonstrate utility but face limitations including assay variability and tumor heterogeneity. Emerging candidates, such as tumor-infiltrating lymphocytes (TILs), relative eosinophil count (REC), lactate dehydrogenase (LDH), and S100 calcium-binding protein B (S100B), offer additional prognostic or predictive value but require further validation.

This review adds value by integrating and comparing both validated and emerging biomarkers across tumor types and by emphasizing practical considerations for real-world application. Given the narrative format and focus on selected cancers, the scope is inherently limited, and not all emerging biomarkers or real-world implementation data are fully addressed. Methodological limitations and current gaps in biomarker validation are also discussed.

## Introduction and background

Immunotherapy has transformed oncology, offering durable clinical responses in several malignancies, including non-small cell lung cancer (NSCLC), melanoma, and triple-negative breast cancer (TNBC) [1,2]. However, only a subset of patients derives clinical benefit from immune checkpoint inhibitors (ICIs), a class of immunotherapy drugs that restore T-cell-mediated antitumor immunity by blocking inhibitory pathways such as programmed cell death protein-1/programmed death-ligand 1 (PD-1/PD-L1) and cytotoxic T-lymphocyte-associated protein 4 (CTLA-4) [3]. The variability in treatment response and the risk of immune-related adverse events (irAEs) underscore the need for reliable biomarkers to guide therapy selection.

Biomarkers are measurable biological indicators that provide information about the presence, progression, or therapeutic responsiveness of disease. Predictive biomarkers identify patients likely to respond to a specific treatment, while prognostic biomarkers provide information about overall clinical outcomes independent of the therapy received [4]. In the context of cancer immunotherapy, predictive biomarkers are essential for tailoring treatment strategies, reducing unnecessary toxicity, and optimizing clinical outcomes.

An ideal biomarker should be specific, reproducible, clinically accessible, and mechanistically informative. Commonly investigated immunotherapy biomarkers include PD-L1, microsatellite instability (MSI), CTLA-4, and tumor-infiltrating lymphocytes (TILs). However, the predictive accuracy of these biomarkers remains inconsistent, often constrained by tumor heterogeneity, assay variability, and dynamic biomarker expression across tumor sites and disease stages [5].

This narrative review synthesizes current evidence on key predictive biomarkers in cancer immunotherapy, particularly in NSCLC, melanoma, and breast and colorectal cancers. Both validated and emerging markers are discussed, with a focus on their clinical applications, limitations, and potential integration into biomarker-driven precision oncology.

These four cancer types were selected due to their relatively advanced integration of immunotherapy in clinical practice and the availability of biomarker data. While this review emphasizes predictive biomarkers, it also addresses prognostic markers, reflecting disease trajectory regardless of therapeutic intervention. Though not exhaustive, this review highlights real-world challenges in biomarker implementation, including assay variability, cost, accessibility, and integration into clinical algorithms.

## Review

Methodology 

This narrative review was conducted to synthesize clinically relevant evidence on predictive and prognostic biomarkers in cancer immunotherapy across selected solid tumors. A narrative review approach was chosen to enable the thematic analysis of established and emerging biomarkers across tumor types, without the procedural constraints of systematic reviews. A comprehensive literature search was performed using PubMed and Scopus, targeting English-language, peer-reviewed articles. Search terms included the following: "immunotherapy", "cancer biomarkers", "PD-L1", "MSI", "CTLA-4", "TILs", "HER2", "LDH", "S100B", "ctDNA", "TMB", "eosinophils", and "multi-omics". Terms were searched independently without Boolean operators or Medical Subject Headings (MeSH) terms. No date restrictions were applied, and studies published through May 2025 were considered.

Studies were included if they focused on biomarker use in immunotherapy for solid tumors. Exclusion criteria included non-English articles, case reports, preclinical or animal studies, and studies without clinical relevance. Full-text review was conducted for eligible articles, and data were extracted regarding biomarker classification, mechanism, clinical application, and known limitations.

Due to the narrative design, no article count or formal bias assessment was conducted. Data were grouped thematically as predictive or prognostic.

Results

To aid clinical interpretation, biomarkers are categorized as either predictive or prognostic based on their primary utility in immunotherapy. Most cited studies pertain to advanced-stage malignancies, reflecting current clinical indications for ICIs.

Predictive Biomarkers

PD-1 and PD-L1: PD-L1, the ligand for PD-1, is frequently expressed on antigen-presenting cells (APCs) and tumor cells. Its expression is often induced by interferon-gamma within the tumor microenvironment [6]. When PD-L1 binds PD-1, T-cell activation is inhibited, resulting in immune tolerance. ICIs such as pembrolizumab and nivolumab (PD-1 inhibitors) and atezolizumab, durvalumab, and avelumab (PD-L1 inhibitors) disrupt this interaction and restore antitumor immunity [6,7].

PD-L1 is a key biomarker in NSCLC. The KEYNOTE-024 trial showed that patients with PD-L1 expression ≥50% experienced improved outcomes with pembrolizumab versus chemotherapy, with a median overall survival (OS) of 30 months versus 14.2 months (HR: 0.63; 95% CI: 0.47-0.86) [8]. These results led to pembrolizumab's approval as first-line therapy in advanced NSCLC.

However, the CheckMate-026 trial using nivolumab failed to show similar OS or progression-free survival (PFS) advantages [9], highlighting the limitations of PD-L1 as a standalone biomarker due to assay variability and tumor heterogeneity [6,10].

MSI and mismatch repair deficiency (dMMR): MSI and dMMR reflect defects in DNA repair pathways, commonly observed in colorectal cancer, resulting in high mutational burden and neoantigen formation [11,12]. The FDA granted tissue-agnostic approval to pembrolizumab in 2017 based on trials like KEYNOTE-016, KEYNOTE-164, and KEYNOTE-158 [13-15]. MSI-high tumors had a 39.6% overall response rate (ORR) with durable responses in 78% of cases [15]. MSI-H/dMMR testing is now recommended in guidelines by the American Society of Clinical Oncology (ASCO) and National Comprehensive Cancer Network (NCCN) [16,17], though its utility is limited to a subset of patients.

CTLA-4 and relative eosinophil count (REC): CTLA-4 is a negative regulator of T-cell activation targeted by ipilimumab in melanoma and other cancers [18]. Recent studies suggest REC, a peripheral blood parameter, may predict CTLA-4 response. In one study, melanoma patients with REC ≥1.5% had a median OS of 27 months versus 5-7 months for lower counts [19]. However, REC remains investigational pending further validation.

TILs: TILs consist primarily of cytotoxic and helper T cells that infiltrate tumors, reflecting host immune response. High TIL levels in TNBC and HER2-positive breast cancers are associated with improved immunotherapy response and prognosis [20-22]. TILs have been incorporated into Scandinavian breast cancer guidelines and recognized by the European Society for Medical Oncology (ESMO) for their role in early-stage breast cancer [23,24]. Despite the absence of universal scoring standards, TILs are low-cost and reproducible.

Human epidermal growth factor receptor 2 (HER2): HER2 is overexpressed in 20-30% of breast cancers and serves as both a predictive and prognostic biomarker [25-27]. HER2-targeted therapies (e.g., trastuzumab) reduce recurrence by ~50% [27], though resistance remains a challenge.

Circulating tumor DNA (ctDNA): ctDNA consists of tumor-derived DNA fragments in the bloodstream [28]. Tie et al. showed that a ctDNA-guided strategy reduced adjuvant chemotherapy use in stage II colon cancer without compromising recurrence-free survival [29]. Bartolomucci et al. reported ctDNA detection in >90% of metastatic cases, with 5-11 months' lead time for relapse [28]. A systematic review by Al-Showbaki et al. found that ≥50% ctDNA reduction within 6-16 weeks post-ICI therapy correlated with better PFS and OS [30], a finding supported by meta-analysis across multiple tumor types [31].

Tumor mutational burden (TMB): TMB, the number of somatic mutations per megabase, reflects neoantigen load and immunogenicity [32]. Pembrolizumab was approved for TMB ≥10 mutations/Mb based on KEYNOTE-158, which showed a 29% ORR vs. 6% in low-TMB tumors [33]. Gandara et al. reported that TMB ≥20 mutations/Mb was associated with improved survival across cancers (HR: 0.52; 95% CI: 0.47-0.58) [34].

Multi-omics biomarkers: Multi-omics approaches integrate genomic, transcriptomic, and proteomic data to improve biomarker precision. Bourbonne et al. demonstrated a ~15% improvement in predictive accuracy using multi-omics with machine learning models [35]. Li et al. identified gene clusters associated with durable response to PD-1 blockade [36]. In the Lung-MAP S1400I trial, high CD8⁺GZB⁺ T-cell infiltration predicted better response to nivolumab, while IL-6 and CXCL13 levels were linked to resistance [37].

Prognostic Biomarkers

Lactate dehydrogenase (LDH): LDH reflects tumor metabolic activity and burden. Elevated LDH is associated with poor prognosis and included in the American Joint Committee on Cancer (AJCC) staging for melanoma [38-40]. High LDH predicts reduced OS with ICIs but lacks predictive value [20,41].

S100 calcium-binding protein B (S100B): S100B is a calcium-binding protein linked to melanoma progression. Elevated levels correlate with poor prognosis [25,42]. Wagner et al. found that patients with S100B >0.3 μg/L and LDH >1.5× upper limit of normal had reduced OS during pembrolizumab treatment [43]. Though widely used in Europe, S100B has limited predictive value and minimal adoption outside select regions.

A structured summary is presented in Table 1.

**Table 1:** Classification and clinical utility of predictive and prognostic biomarkers in cancer immunotherapy PD-1: programmed cell death protein-1; PD-L1: programmed death-ligand 1; MSI-H: microsatellite instability-high; dMMR: deficient mismatch repair; LDH: lactate dehydrogenase; CTLA-4: cytotoxic T-lymphocyte-associated protein 4; REC: relative eosinophil count; S100B: S100 calcium-binding protein B; HER2: human epidermal growth factor receptor 2; TILs: tumor-infiltrating lymphocytes; ctDNA: circulating tumor DNA; TMB: tumor mutational burden; NSCLC: non-small cell lung cancer; TNBC: triple-negative breast cancer; RCC: renal cell carcinoma; ICIs: immune checkpoint inhibitors

Biomarker	Type	Mechanism of action	Clinical application	Associated cancers	Predictive value	Limitations
PD-L1	Immune checkpoint	Inhibits T-cell activity via binding to PD-1 [6,7]	Selection for PD-1/PD-L1 inhibitors [8-10]	NSCLC, melanoma, TNBC	Moderate	Inconsistent results; assay variability [10]
MSI-H/dMMR	Genomic instability	Produces neoantigens via DNA repair defects [11,13]	Tissue-agnostic FDA approval for ICIs [14-16]	Colorectal, endometrial, gastric	High	Rare across most cancers [15]
LDH	Serum marker	Reflects tumor burden and metabolism [38,39]	Prognostic marker; treatment monitoring [40,41]	Melanoma, RCC	Low	No validated predictive assay [41]
CTLA-4	Immune checkpoint	Suppresses early T-cell activation [18]	Targeted by ipilimumab [18,21]	Melanoma, RCC	Low	Not predictive; no standardized assay [21]
REC	Peripheral blood marker	May reflect immune activation in response to ICIs [19]	Emerging predictor of CTLA-4 inhibitor response [19]	Melanoma	Moderate	Requires validation in larger cohorts [19]
S100B	Protein marker	Marker of melanoma cell death and disease burden [42]	Disease surveillance in melanoma [42,43]	Melanoma	Low	Prognostic only; limited global adoption [43]
HER2	Oncoprotein	Promotes tumor growth via PI3K/AKT pathway [26]	Selection for HER2-targeted therapies [27,28]	Breast, gastric	High	Not predictive of immunotherapy response [26]
TILs	Immune tissue marker	Indicates immune infiltration in tumor microenvironment [20]	Prognostic and predictive use in breast cancer [21-24]	TNBC, HER2-positive breast cancer, melanoma	High	No universal scoring standard; interpretive variability [24]
ctDNA	Liquid biopsy	Reflects tumor mutational landscape and burden [28]	Monitoring response, detecting minimal residual disease [28,29]	Colorectal, NSCLC, breast, melanoma	High	Still under validation; detection sensitivity varies [30]
TMB	Genomic marker	Indicates neoantigen load and immune responsiveness [32]	Patient selection for ICIs; FDA-approved for TMB ≥10 mut/Mb [33]	NSCLC, melanoma, urothelial carcinoma	High	Thresholds vary by assay; not predictive in all cancers [34]
Multi-omics	Integrated biomarker platform	Combines genomic, transcriptomic, proteomic data [35]	Experimental predictive modeling; stratification [35-37]	NSCLC, melanoma (research-based)	High (investigational)	Requires validation; complex implementation [35-37]

Discussion****

The integration of biomarkers into cancer immunotherapy represents one of the most promising advancements in precision oncology. These biomarkers offer clinicians the ability to identify patients most likely to benefit from ICIs while sparing others from unnecessary toxicity and cost. However, despite their promise, current biomarkers face several limitations in predictive accuracy, accessibility, and applicability across diverse clinical settings.

Assay variability and tumor heterogeneity are two of the most significant challenges limiting biomarker generalizability. For example, PD-L1 expression is highly dependent on the antibody clone used, the scoring system applied, and the tumor sampling site, all of which contribute to inconsistent results across studies [7]. In the KEYNOTE-024 trial, PD-L1 expression ≥50% strongly predicted benefit from pembrolizumab in patients with NSCLC, with significantly improved OS compared to chemotherapy [8]. Consequently, PD-L1 testing has been incorporated into major clinical practice guidelines, including those from the NCCN and the ESMO, to guide ICI use in NSCLC [44]. In contrast, the CheckMate-026 trial, which evaluated nivolumab in a similar patient population, failed to demonstrate clinical benefit based on PD-L1 expression alone [9]. This inconsistency underscores the combined impact of assay variability and intertumoral heterogeneity in PD-L1 assessment.

Similarly, HER2 testing varies depending on whether immunohistochemistry (IHC) or in situ hybridization (ISH) platforms are used, affecting both treatment eligibility and therapeutic outcomes in breast and gastric cancers [25,26]. The absence of universal assay standardization and interpretation criteria limits the reproducibility of biomarker assessments and undermines their clinical reliability. These examples highlight the need for harmonized testing protocols and validated thresholds to improve biomarker consistency and utility.

The distinction between blood-based and tissue-based biomarkers carries important clinical implications. Blood-based markers such as LDH, S100B, and REC offer practical advantages, including accessibility, affordability, and the ability to monitor patients longitudinally during treatment [19,38,43,45]. However, these biomarkers often suffer from reduced specificity and weaker mechanistic links to tumor-immune interactions when compared to tissue-based biomarkers such as TILs or PD-L1 expression via IHC [6,20,21]. Although tissue biomarkers offer deeper biological insight, they require invasive procedures and are prone to sampling bias due to intratumoral heterogeneity [7,21]. A multimodal approach that integrates both blood- and tissue-derived data may therefore provide a more comprehensive and clinically feasible strategy for immunotherapy selection.

Real-world implementation challenges, particularly in low-resource settings, further hinder biomarker adoption. In many countries, infrastructure to support advanced IHC or next-generation sequencing (NGS) for biomarkers such as PD-L1, MSI-H, or TMB is lacking [13,14,46]. Even in high-income settings, cost and reimbursement limitations often restrict routine use. For example, S100B is commonly employed in Germany for melanoma surveillance, but its limited global validation has precluded widespread use [43]. Likewise, TILs have been incorporated into national breast cancer guidelines in countries such as Sweden and Denmark, yet broader adoption is limited by interobserver variability and a lack of standardized scoring frameworks [23]. These disparities emphasize the urgent need for globally accessible, low-cost, and scalable biomarker platforms, especially in resource-limited health systems.

Recent advances in circulating biomarkers such as ctDNA, TMB, and multi-omics panels have further expanded the biomarker landscape. ctDNA has demonstrated utility in real-time treatment monitoring and early relapse detection across multiple tumor types [28-31]. TMB ≥10 mutations per megabase has been used as the basis for FDA approval of pembrolizumab in tissue-agnostic indications [33]. These markers are now being integrated into clinical guidelines and regulatory frameworks [14,15,33]. Moreover, multi-omics approaches, combining genomic, transcriptomic, and proteomic data, have shown improved predictive performance when aided by machine learning models [35-37]. However, these platforms remain investigational and require validation in large-scale prospective trials.

Looking ahead, artificial intelligence (AI) and machine learning are poised to play a transformative role in biomarker-guided immunotherapy. By integrating high-dimensional data from genomics, transcriptomics, proteomics, and radiomics, AI platforms can identify complex biomarker signatures that surpass the limitations of univariate models. Recent work by Camps et al. illustrates how AI-driven integration of multi-omics and radiomics, including liquid biopsy, ctDNA, and imaging features, can improve diagnostic precision and response monitoring [12,47]. Their mini-review further highlights the potential of single-cell and spatial transcriptomics, alongside radiomic and pathomic data, to reveal tumor heterogeneity and optimize patient stratification for immunotherapy. Although data harmonization and clinical implementation remain challenges, AI also holds promise for predicting irAEs using longitudinal blood-based data, as shown in emerging deep learning models [48]. While these strategies are largely investigational, they offer substantial potential for developing adaptive, individualized biomarker panels to enhance treatment personalization and monitoring.

Limitation****

While this review provides a comprehensive overview of predictive biomarkers in cancer immunotherapy, several limitations must be acknowledged. First, the narrative nature of the review introduces a potential risk of selection bias, as it lacks the systematic methodology employed in meta-analyses. Second, only peer-reviewed literature published in English was included, which may exclude relevant findings from non-English or unpublished sources. Third, preclinical data and early-phase studies were not emphasized, which could limit insight into the next wave of biomarker candidates. Additionally, heterogeneity in assay platforms and interpretation across different studies complicates direct comparison of biomarker efficacy. Finally, real-world data, particularly from low-resource settings, remain underrepresented in the current evidence base, thereby affecting generalizability.

## Conclusions

The integration of predictive and prognostic biomarkers into cancer immunotherapy represents one of the most promising advances in precision oncology. These biomarkers provide clinicians with tools to predict treatment responsiveness, stratify risk, and monitor disease trajectory. However, despite the promise of emerging markers, their predictive and prognostic utility must be interpreted cautiously. Many remain investigational and lack validation in large, diverse patient cohorts or across different tumor types. As such, the conclusions drawn in this review reflect the current state of evidence and deliberately avoid overstating the clinical readiness of novel biomarkers.

Assay variability, lack of standardization, and tumor heterogeneity continue to limit the reproducibility and reliability of biomarker results. Addressing these limitations will require harmonization of testing protocols, universal scoring systems, particularly for PD-L1, TILs, and HER2, and greater methodological rigor in biomarker validation.

Looking ahead, future research must prioritize large-scale prospective trials across diverse populations to validate biomarker performance and applicability. Specific attention should be given to refining blood-based markers for ease of use and improving cross-platform comparability for tissue-based assays. The development of clear thresholds, scoring systems, and interpretation guidelines will be essential for translating emerging biomarkers into routine clinical use.

The concept of integrated biomarker panels, incorporating multi-omics data and aided by AI, holds significant theoretical promise. Early research supports their potential, particularly in identifying composite signatures that outperform single-variable models. However, these approaches are still in exploratory stages and must undergo clinical validation before adoption into practice.

In summary, while no single biomarker currently meets all the criteria for universal application in immunotherapy, a multimodal and interdisciplinary approach, grounded in clinical evidence and mindful of real-world constraints, offers the most viable path forward for advancing precision immuno-oncology.
